# scPEDSSC: proximity enhanced deep sparse subspace clustering method for scRNA-seq data

**DOI:** 10.1371/journal.pcbi.1012924

**Published:** 2025-04-28

**Authors:** Xiaopeng Wei, Jingli Wu, Gaoshi Li, Jiafei Liu, Xi Wu, Chang He

**Affiliations:** 1 Guangxi Key Lab of Multi-source Information Mining & Security, Guangxi Normal University, Guilin, Guangxi, China; 2 College of Computer Science and Engineering, Guangxi Normal University, Guilin, Guangxi, China; 3 Key Lab of Education Blockchain and Intelligent Technology, Ministry of Education, Guangxi Normal University, Guilin, Guangxi, China; North Carolina State University, UNITED STATES OFAMERICA

## Abstract

It is a significant step for single cell analysis to identify cell types through clustering single-cell RNA sequencing (scRNA-seq) data. However, great challenges still remain due to the inherent high-dimensionality, noise, and sparsity of scRNA-seq data. In this study, scPEDSSC, a deep sparse subspace clustering method based on proximity enhancement, is put forward. The self-expression matrix (SEM), learned from the deep auto-encoder with two part generalized gamma (TPGG) distribution, are adopted to generate the similarity matrix along with its second power. Compared with eight state-of-the-art single-cell clustering methods on twelve real biological datasets, the proposed method scPEDSSC can achieve superior performance in most datasets, which has been verified through a number of experiments.

## Author summary

The rapid advancement of single-cell RNA sequencing technologies has thrown a new light on studying complex biological phenomena. A crucial step in the single-cell transcriptome analysis is to group cells which belong to the same cell type with gene expression data, i.e., clustering a noisy, sparse and high dimensional dataset with enormously fewer cells than the number of genes. In order to address the above problems, we propose a deep sparse subspace clustering method based on proximity enhancement. The raw sequencing data are first preprocessed by four different similarities and the corresponding Laplace scores to initially reduce their dimensionality. Afterwards, the self-expression matrix (SEM), learned from the deep auto-encoder with two part generalized gamma (TPGG) distribution, are adopted to generate the similarity matrix along with its second power. The clustering results are finally obtained using spectral clustering. Experimental comparisons with eight state-of-the-art methods on multiple datasets demonstrate the effectiveness and reliability of method scPEDSSC in clustering scRNA-seq data.

## Introduction

Single-cell RNA sequencing (scRNA-seq) is an emerging high-throughput sequencing technology. It can overcome inherent defects of traditional sequencing, unable to reflect the actual situation of each cell due to averaging the expression of cell groups, by detecting the gene expression status at the single-cell resolution [1,2]. ScRNA-seq technology can provide significant support and assistance to explore intercellular heterogeneity and gain insight into biological processes [3]. Cell type identification is one of the fundamental upstream tasks for conducting these studies [4], hence it is essential to differentiate varieties of cells from scRNA-seq data. Great attention has been drawn to devise new efficient and reliable clustering methods, for the traditional ones cannot deal with high noise rate and high dropouts inherent in scRNA-seq data [5–7].

It has been acknowledged that deep learning approaches can provide a unique opportunity to model the noisy and complex scRNA-seq data [8]. In recent years, many deep learning-based cluster methods have been put forward. In 2019, Tian et al. [9] proposed the scDeepCluster method, which adds Gaussian noise to each coding layer and applies deep embedding clustering to generate final cell clusters. In 2022, method scBKAP presented by Wang et al. [10] conducted bisecting *K*-means clustering on the dimensionality-reduced single-cell data, which were generated from an autoencoder network and a dimensionality reduction model MPDR. In 2023, Du et al. [11] proposed a self-supervised contrastive learning method scCCL for clustering scRNA-seq data, which uses momentum encoder to extract features from enhanced data, and implements contrast learning in the instance-level and cluster-level modules to obtain a higher-order embedding representation model. In the same year, He et al. [12] put forward method scMCKC, which performs denoising and dimensionality reduction with a zero-inflated negative binomial model-based autoencoder, and conducts a weighted soft *K*-means clustering on latent space by using the pairwise constraints with a priori information.

Since the high noise existed in scRNA-seq data makes it challenging to explore group structure in high dimensional space, subspace clustering has been adopted to capture global structural information and yield more reliable similarity [8]. In 2019, Zheng et al. [13] proposed a similarity learning-based method SinNLRR, which learns non-negative and low-rank constrained similarity matrices for the purpose of dimensionality reduction and clustering. In 2021, Liang et al. [14] devised method SSRE, which computes the linear representation between cells based on sparse subspace theory, and generates a sparse representation of the cell-to-cell similarity. Later, Wang et al. [8] indicated that the subspace-based models ignored the abundant distribution and manifold information contained in scRNA-seq data, i.e., the learnt feature representation can not thoroughly imply the deep relationships of subspaces. The scDSSC method proposed by them combines noise reduction and dimensionality reduction for scRNA-seq data, modelling scRNA-seq data with a zero-inflated negative binomial (ZINB) distribution, and constructing the similarity matrix from the learned hidden layer self-expression one. However, a recent study [15] has indicated that the normalized scRNA-seq data exhibit such two statistical features as the bimodal expression pattern and the right-skewed characteristic, which may not be modeled by the ZINB distribution. In this paper, the two part generalized gamma (TPGG) distribution is introduced for modeling the scRNA-seq data with such statistical features. The main contributions are as follows:

1. Devise a deep auto-encoder by introducing the two part generalized gamma distribution to better extract the features of the gene expression matrix.
2. Explore the potential relationships between cells by conducting the calculation of their second-order proximity, making the self-expression matrix contain more comprehensive information between cells.
3. Propose a Proximity Enhancement based Deep Sparse Subspace Clustering method (scPEDSSC) to cluster cells with scRNA-seq Data. It constructs the similarity matrix with the enhanced hidden layer self-expression one, and then performs spectral clustering on it to acquire cell clusters.
4. Extensive comparative trials were conducted on twelve real datasets, the results prove the effectiveness of the proposed method compared to the state-of-the-art approaches.

## Materials and methods

Suppose that there is an *m* × *n* gene expression matrix *X*, where the rows denote a group of different types of cells *C*, the columns denote a set of genes *G*, and each entry *x_ij_* represents the expression level of gene *j* in cell *i* (*i*=1, 2, …, *m*, *j*=1, 2, …, *n*). The cell clustering method tries to partition the *m* cells into a set of *K* clusters, i.e.,

$$\{C_{1},C_{2},\ldots ,C_{K}|C_{i}\underset{i\neq j}{⋂}C_{j}=\emptyset ,\overset{K}{\underset{i=1}{⋃}}C_{i}=C\},$$

so that the same type of cells can be categorized into the same cluster.

**Fig 1:**
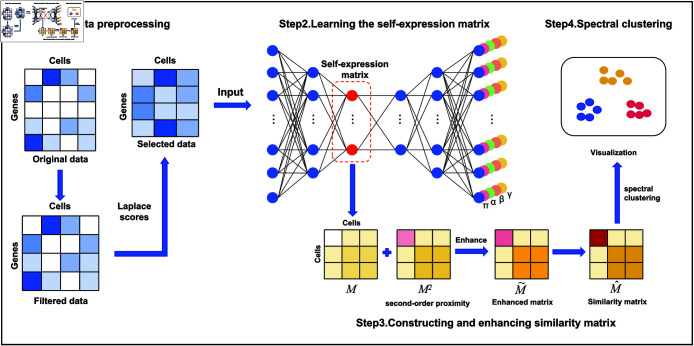
Fig 1 The pipeline of method scPEDSSC. Step 1: Data preprocessing. Step 2: Learning the self-expression matrix. Step 3: Constructing and enhancing similarity matrix. Step 4: Spectral clustering.

Based on the above notations and definitions, a novel deep sparse subspace clustering method scPEDSSC is put forward. As shown in Fig 1, we begin with preprocessing the original gene expression data, i.e., droping the genes that are not expressed in all cells, and selecting a given number of genes with high Laplace scores. Then a self-expression matrix is generated from training a deep auto-encoder with preprocessed gene expression data. Next, a similarity matrix is constructed from the self-expression one enhanced with its second-order proximity. Finally, a spectral clustering is conducted to produce a group of clusters. Some critical techniques of method scPEDSSC are described as follows.

### Data preprocessing

Since low-expressed genes fail to provide valid information for clustering in most cases, they are filtered out from the given gene expression matrix *X* so as to reduce the dimensionality of the data [16–18]. We begin with dropping the genes that are not expressed in all of the cells. Then each row is normalized with *L*2 norm to eliminate the expression scale differences between cells. Next, four gene-gene similarity matrices $M_{s}$, $M_{p}$, $M_{sp}$, and $M_{c}$ are created with calculating such four correlation coefficients as Sparse, Pearson, Spearman, and Cosine on the normalized expression matrix [14]. For each gene, four Laplace scores are computed based on the four similarity matrices. Finally, the top *T* genes with higher harmonic mean of the four Laplace scores are retained. For the convenience of description, the preprocessed gene expression matrix is still denoted by *X*.

### Learning the self-expression matrix

Due to the limitations of the sequencing technique, the scRNA-seq data are represented with high sparsity. Therefore, the theory of sparse subspace [19], an approach for uncovering the internal structure of complex data in an unsupervised manner, is applied to represent the similarity between cells. The calculation of self-expression matrix is a critical step in clustering, i.e., the expression profile of a cell is mathematically described as a linear combination of the expression profiles of the cells predicted to be the same type [8]. It is able to capture global structural information and create more reliable similarity. Nevertheless, it is a challenging task to extract robust descriptive features from the high dimensional scRNA-seq data. In this section, a deep autoencoder neural network is constructed to project them into a low dimensional space, so as to acquire the low dimensional representations with rich non-linear features. As illustrated in Step 2 of Fig 1, two three-layer fully-connected neural networks are adopted as encoder and decoder, with $n_{i}^{e}$ and $n_{i}^{d}$ (*i*=1, 2, 3) neurons on the *i*th layer of the encoder and decoder, respectively. The hidden layer, extracted from the preprocessed expression matrix through the encoder, is adopted to calculate the self-expression matrix. The loss function can be formulated as follows:

$$\begin{array}{ccc} \begin{array}{ccc} & \underset{L}{\min}L_{rescon}+L_{self}+L_{spar}, & \\ & L_{rescon}=\frac{1}{2}∥X-\hat{X}{∥}_{F}^{2},\\ & L_{self}=\frac{1}{2}∥Z-ZM{∥}_{F}^{2},\\ & L_{spar}=∥M{∥}_{F}^{2}, \end{array} & & \end{array}$$ (1)

$$\begin{array}{ccc} s.t.diag(M)=0,Z=E(X),\hat{X}=D(Z), & & \end{array}$$ (2)

where $\hat{X}$ denotes the reconstructed data, *M* is the self-expression matrix, *E*( ⋅ ) and *D*( ⋅ ) represent two nonlinear mapping, i.e., the encoding and decoding process, *Z* is the low-dimensional embedding features. The term $∥M{∥}_{F}^{2}$ imposes sparsity restriction on the self-expression matrix.

It is crucial to select an appropriate probability distribution function to model the distributional properties of scRNA-seq data. The ZINB distribution has been applied in most models [8,18,20], for its good simulation of the sparsity of single-cell data. However, it is discovered that the non-zero values in normalized scRNA-seq data usually present such two features as bimodality and right-skew [15,21], which are neglected by the ZINB distribution. Therefore, in this study, the TPGG distribution [15] that takes the two features into full consideration is employed. As shown in Fig 1, additional four fully-connected layers (denoted with four different colors) are applied in the decoder, trying to simulate the TPGG distribution, as represented in Eq (3):

$$\begin{array}{ccc} f_{TPGG}(x\;|\;\pi ,\;\alpha ,\;\beta ,\;\gamma )\;=\;\pi I_{\left[x=0\right]}\;+\;(1\;-\;\pi )I_{\left[x>0\right]}f_{G}(x\;|\;\alpha ,\;\beta ,\;\gamma ), & & \end{array}$$ (3)

where *π* (*π* ∈ [0,1]) is the parameter of Bernoulli distribution, fitting for the probability of observing a positive-versus-zero outcome. *α*, *β*, and *γ* (*α* > 0, *β* > 0, *γ* > 0) are the shape and scale parameters of the generalized gamma distribution, as shown in Eq (4):

$$\begin{array}{ccc} f_{G}(x|\alpha ,\beta ,\gamma )=\frac{\gamma }{\Gamma (\beta )}\frac{x^{\beta \gamma -1}}{\alpha^{\beta \gamma }}e^{-(\frac{x}{\alpha })\gamma }, & & \end{array}$$ (4)

here *Γ*( ⋅ ) denotes the gamma function. As indicated in Fig 1, the autoencoder is utilized to estimate the four parameters, which are set as the decoder outputs through four fully connected layers. The rules of forward propagation is illustrated as follows:

$$\begin{array}{ccc} \begin{array}{ccc} H_{l} & =\sigma (H_{l-1}W_{l-1}),(l=1,2,\ldots ,D-1), & \\ \Pi & =sigmoid(H_{D-1}W_{D_{\pi }}),\\ A & =softplus(H_{D-1}W_{D_{\alpha }}),\\ B & =softplus(H_{D-1}W_{D_{\beta }}),\\ Y & =softplus(H_{D-1}W_{D_{\gamma }}). \end{array} & & \end{array}$$ (5)

In Eq (5), the first equation represents the process of forward propagation, where *D*–1 denotes the penultimate layer of the decoder network. $H_{0}$ denotes the preprocessed gene expression matrix *X*. *σ*( ⋅ ) is the activation function, and the ReLU function is used here. *W* denotes the weight matrix. *Π*, *A*, *B*, and *Y* represent four inferred parameter matrices outputted by the decoder. Then the negative log-likelihood of TPGG is used to construct the loss function, connecting the inputs and outputs efficiently, as follows:

$$L_{TPSS}=-\overset{m}{\underset{i=1}{\sum }}\overset{n}{\underset{j=1}{\sum }}\log(f_{TPSS}(x_{ij}|\pi_{ij},\alpha_{ij},\beta_{ij},\gamma_{ij}))+\underset{t\in S}{\sum }∥W_{t}{∥}_{F}^{2},$$ (6)

where *S* denotes set {0,  ⋯ , *D*–2, $D_{\pi }$, $D_{\alpha }$, $D_{\beta }$, $D_{\gamma }$}. The regularization term attempts to prevent the effect of static noise on the optimization objective and the irrelevant components of learnable parameters. Thus, the final loss function of the presented model is formulated as below:

$$\begin{array}{ccc} L={\left(\lambda_{1}L_{rescon}+\lambda_{2}L_{self}+\lambda_{3}L_{spar}\right)}^{\frac{1}{10}}+L_{TPSS}, & & \end{array}$$ (7)

here $\lambda_{1}$, $\lambda_{2}$, $\lambda_{3}$ are there hyperparameters. Based on the loss function *L*, the model is trained with learning rate *lr*.

### Constructing similarity matrix from enhanced self-expression one

As mentioned above, although the learned self-expression matrix is able to capture the global structural information among cells, some inherent higher-order relations [22] remain unextracted. Therefore, enhancement was performed on self-expression matrix by executing its second power. Let matrix *M* be the learned *m* × *m* self-expression matrix, where both rows and columns represent cells, each entry *M*[*i*,*j*] measures the relationship from the *i*-th cell to the *j*-th one. The intuition of performing second power is that the direct relationship from cell *i* to cell *j* may be enhanced through the transitivity of relationships. The relationship is also proportional to the number intermediary cells transiting relationships and the strength of the relationships with the intermediary ones. Let $\tilde{M}$ denote the enhanced matrix, where $\tilde{M}$[*i*,*j*] (*i*, *j*=1, 2, …, *m*) is calculated as Eq (8):

$$\begin{array}{ccc} \tilde{M}[i,j]=\{\begin{array}{cc} \overset{m}{\underset{k=0}{\sum }}M[i,j]+M[i,k]\times [k,j]\; & \text{if}i\neq j,\\ 0\; & \text{otherwise}. \end{array} & & \end{array}$$ (8)

**Fig 2:**
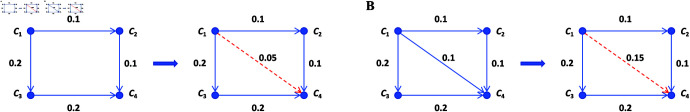
An example of proximity enhancement.

Let us take Fig 2 for an example, there are some relationships, denoted with directed edges, among cells $c_{1}$, $c_{2}$, $c_{3}$ and $c_{4}$. In Fig 2A, a potential direct relationship may be created between cells $c_{1}$ and $c_{4}$ through intermediary cells $c_{2}$ and $c_{3}$. Then its strength is set to 0 . 0 + 0 . 1 × 0 . 1 + 0 . 2 × 0 . 2 = 0 . 05. Similarly, in Fig 2B, the strength of relationship between cells $c_{1}$ and $c_{4}$ is updated to 0 . 1 + 0 . 1 × 0 . 1 + 0 . 2 × 0 . 2 = 0 . 15.

Given the enhanced self-expression matrix $\tilde{M}$, the similarity matrix $\hat{M}$ is constructed as follows:

$$\begin{array}{ccc} \hat{M}=|\tilde{M}|+|{\tilde{M}}^{T}|. & & \end{array}$$ (9)

### Spectral clustering

Given the constructed similarity matrix $\hat{M}$, spectral clustering, which has the advantage of model simplicity and robustness, is adopted to cluster the cells. It begins with decomposing similarity matrix $\hat{M}$ with Singular Value Decomposition (SVD) algorithm, and normalizing the left singular vector with *L*2 norm and max norm. Let $M_{l}$ denote the normalized left singular vector, the matrix $[(M_{l}+M_{l}^{T})]∕2$ is obtained and still denoted as $\hat{M}$ for the convenience of description. Then the Laplace matrix *L = D − A_M_* is constructed to acquire its eigenvalues and eigenvectors, where *A_M_* is the adjacent matrix generated by performing the *K*-Nearest Neighbor (KNN) algorithm on matrix $\hat{M}$ (*K* = 10)[8], *D* is the degree matrix. Finally, K-means algorithm is employed to acquired the clustered cells, where the number of clusters is set to the actual number of labels. The detailed illustration of spectral clustering could refer to previous literature [23,24].

## Results

In this section, real scRNA-seq datasets were adopted to compare the performance of method scPEDSSC with eight state-of-the-art methods: two traditional methods NMF [6] and SIMLR [7], four deep learning-based methods scCCL [11], scBKAP [10], scMCKC [12], and scDCC [25], two subspace clustering methods SSRE [14] and scDSSC [8]. The source code of the comparison methods was acquired from the literature. All of the experiments were conducted on an Intel Core i7-12700 2.10 GHz with 16GB RAM. The operating system was Windows 11, and the deep learning framework was TensorFlow 1.2.1 for method scBKAP, and PyTorch 3.8 for the other methods.

### Datasets

Twelve real scRNA-seq datasets were collected from public databases or published studies. The number of cells ranges from hundreds to thousands, and the number of genes are from thousands to tens of thousands. The details of the datasets are exhibited in Table 1.

**Table 1:** The details of real scRNA-seq datasets adopted in the experiments.

Dataset	Cell number	Gene number	Cell types
Ting [26]	114	14405	5
Goolam [27]	124	40315	5
Deng [28]	135	12548	7
Engel4 [29]	203	23337	4
Song [30]	214	27473	4
Pollen [31]	301	23730	11
Darmanis [32]	420	22085	8
Haber [33]	1522	20108	9
Tasic [34]	1727	5832	48
Vento [35]	5418	33693	38
HumanLiver [25]	8444	5000	11
CITE_CBMC [36]	8617	2000	15

### Evaluation metrics and parameters settings

As performed in previous studies [8,14,15,25], two widely used evaluation metrics, i.e., Adjusted Rand Index (ARI) [37] and Normalized Mutual Information (NMI) [38], were adopted to quantitatively evaluate the clustering performance. Both of them evaluate the performance of clustering by assessing the similarity between genuine class labels and predicted cluster ones. The larger they are, the better a clustering result is. Given a group of *m* cells *C*, let $P_{1}$={$P_{11}$,$P_{12}$,…,$P_{1k_{1}}$} denote the genuine partition of *C* into $k_{1}$ subsets, let $P_{2}$={$P_{21}$,$P_{22}$,…,$P_{2k_{2}}$} denote the predicted partition of *C* into $k_{2}$ subsets. The calculation of ARI is as Eq (10):

$$\begin{array}{ccc} ARI(P_{1},P_{2})=\frac{2(ad-bc)}{\left(a+b)(b+d)+(a+c)(c+d\right)}, & & \end{array}$$ (10)

where *a* represents the number of pairs of cells in *C* that are in the same subset in $P_{1}$ and $P_{2}$. *b* denotes the number of pairs of cells in *C* that are in the same subset in $P_{1}$ but in different subsets in $P_{2}$. *c* equals the number of pairs of cells in *C* that are in different subsets in $P_{1}$ but in same subset in $P_{2}$. *d* denotes the number of pairs of cells in *C* that are in different subsets in $P_{1}$ and $P_{2}$. NMI is calculated as in Eq (11):

$$\begin{array}{ccc} NMI(P_{1},P_{2})=\frac{2MI(P_{1},P_{2})}{H(P_{1})+H(P_{2})}, & & \end{array}$$ (11)

$$MI(P_{1},P_{2})=\overset{k_{1}}{\underset{i=1}{\sum }}\overset{k_{2}}{\underset{j=1}{\sum }}p(i,j)\log\frac{p(i,j)}{p(i)p(j)},$$ (12)

$$\begin{array}{ccc} H(P_{1})=-\overset{k_{1}}{\underset{i=1}{\sum }}p(i)\log p(i), & & \end{array}$$ (13)

$$\begin{array}{ccc} H(P_{2})=-\overset{k_{2}}{\underset{j=1}{\sum }}p(j)\log p(j), & & \end{array}$$ (14)

where *MI*($P_{1}$,$P_{2}$) represents mutual information of $P_{1}$ and $P_{2}$, *H*($P_{1}$) (resp. *H*($P_{2}$)) represents the entropy of $P_{1}$ (resp. $P_{2}$). *p*(*i*)=$\frac{\left|P_{1i}\right|}{m}$, *p*(*j*)=$\frac{\left|P_{2j}\right|}{m}$, and *p*(*i*,*j*)=$\frac{\left|P_{1i}\cap P_{2j}\right|}{m}$.

The parameters of method scPEDSSC were set as follows: *T* = 2000, $\lambda_{1}=0.2$, $\lambda_{2}=1.0$, $\lambda_{3}=0.5$, $n_{1}^{e}$ = $n_{3}^{d}$ = 256, $n_{2}^{e}$ = $n_{2}^{d}$ = 32, $n_{3}^{e}$ = $n_{1}^{d}$ = 10, and $l_{r}$ = 0.001, which were ascertained through a large number of experimental tests, as shown in S1 and S2 Tables. The parameters of the other methods were set as the literature[6–8,10–12,14,25].

### Cell type identification and analysis by clustering

In Table 2, the scPEDSSC method is compared with other methods based on the Normalized Mutual Information. During the experiments, the number of clusters is set to the actual number of labels, i.e., $k_{2}$=$k_{1}$. The last row AVG_Rank indicates the average rank among the comparative methods. It has the same meaning in the subsequent table. A smaller AVG_Rank means better performance. As can be seen from the table, the proposed method scPEDSSC has achieved the best results on half of the datasets except for Ting (ranked 2nd), Deng (ranked 2nd), Vento (ranked 2nd), CITE_CBMC (ranked 2nd), Tasic (ranked 3rd) and HumanLiver (ranked 3rd). It has earned average rank of 1.6, indicating it performs superior to the other methods in general.

**Table 2:** Comparison of NMI for the twelve real datasets.

Datasets	NMF	SIMLR	scCCL	scBKAP	scMCKC	scDCC	scDSSC	SSRE	scPEDSSC
Ting	0.845	0.900	0.740	0.877	0.709	0.746	0.783	**1.000**	0.949
Goolam	0.572	0.731	0.742	0.683	0.789	0.661	0.601	0.829	**0.878**
Deng	0.605	0.639	0.766	0.743	0.717	0.726	0.637	**0.813**	0.785
Engel4	0.475	0.734	0.251	0.627	0.767	0.598	0.401	0.773	**0.774**
Song	0.027	0.673	0.715	0.713	0.477	0.698	0.561	0.733	**0.763**
Pollen	0.917	0.771	0.904	0.918	0.889	0.866	0.911	0.931	**0.946**
Darmanis	0.036	0.591	0.590	0.561	0.610	-	0.652	0.814	**0.861**
Haber	0.011	0.417	0.571	0.143	0.616	-	0.656	0.532	**0.669**
Tasic	0.378	0.465	0.410	0.420	0.349	0.342	0.417	**0.474**	0.454
Vento	0.098	0.678	0.620	0.577	0.560	0.633	0.588	**0.719**	0.681
HumanLiver	0.003	0.668	0.668	-	0.816	**0.858**	0.567	0.695	0.795
CITE_CBMC	0.005	0.674	0.459	-	0.752	**0.768**	0.463	0.635	0.765
AVG_Rank	7.6	5	5.6	5.8	5.5	5.9	6	2.3	**1.6**

Table 3 illustrates the comparison results in terms of the Adjusted Rand Index. It can be observed that method scPEDSSC still performs the best on most (seven out of twelve) datasets, and its smallest AVG_Rank demonstrates that it has better performance in general than other comparison methods.

**Table 3:** Comparison of ARI for the twelve real datasets.

Datasets	NMF	SIMLR	scCCL	scBKAP	scMCKC	scDCC	scDSSC	SSRE	scPEDSSC
Ting	0.735	0.871	0.682	0.862	0.541	0.500	0.678	**1.000**	0.946
Goolam	0.404	0.608	0.790	0.517	0.644	0.440	0.559	0.668	**0.885**
Deng	0.356	0.384	0.589	0.477	0.524	0.525	0.379	0.650	**0.729**
Engel4	0.369	0.683	0.234	0.585	0.660	0.439	0.380	**0.755**	0.748
Song	0.008	0.674	0.665	0.674	0.371	0.607	0.569	0.748	**0.782**
Pollen	0.866	0.501	0.880	0.927	0.807	0.735	0.861	0.888	**0.932**
Darmanis	0.002	0.401	0.527	0.440	0.571	-	0.615	0.712	**0.886**
Haber	0.001	0.234	0.423	0.043	0.496	-	0.486	0.321	**0.508**
Tasic	0.092	0.125	0.094	0.106	0.080	0.079	0.110	0.131	**0.132**
Vento	0.012	0.463	0.285	0.293	0.272	0.443	0.327	**0.479**	0.440
HumanLiver	0.001	0.355	0.486	-	0.839	**0.878**	0.479	0.441	0.685
CITE_CBMC	0.001	0.564	**0.649**	-	0.643	0.614	0.267	0.427	0.618
AVG_Rank	7.7	5	4.7	5.8	5.1	6.2	5.7	2.8	**1.8**

### Visualization of cell clustering

**Fig 3:**
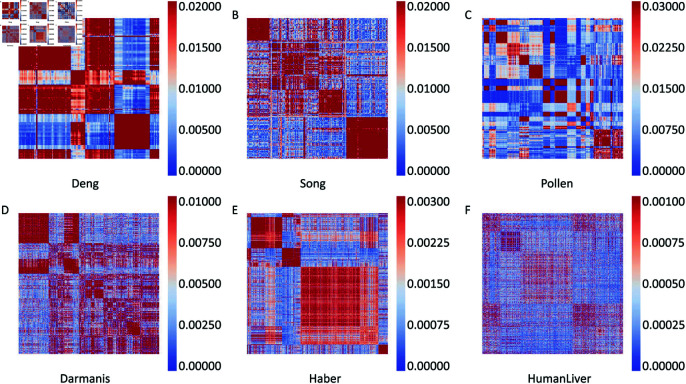
The heatmaps of similarity matrices.

As mentioned above, spectral clustering is applied on the constructed similarity matrix $\hat{M}$, which records the potential correlations among cells. To illustrate more intuitively the relationships, the heatmaps of similarity matrices are exhibited for six datasets with different sizes, as shown in Fig 3. Redder color indicates a stronger correlation, while bluer color indicates a weaker one. From this figure it can be seen that the cells are indeed distributed in different low-dimensional subspaces. The cells belong to the same subspace have strong relationships with each other.

**Fig 4:**
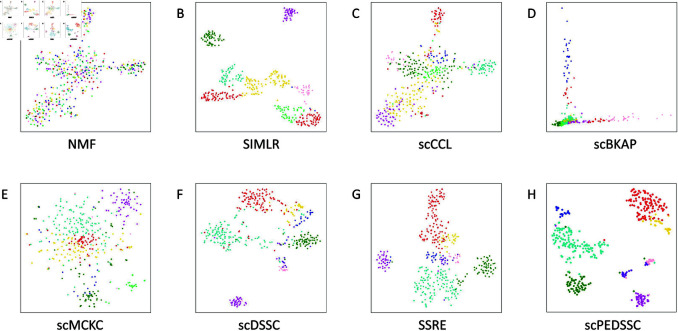
Visual comparisons of clustering results on the Darmanis dataset.

In Fig 4, the clustering results of the comparison methods on the Darmanis dataset was visually compared using scatter plots. Specifically, t-distributed Stochastic Neighbor Embedding (t-SNE), a popular dimensionality reduction and visualization technique, was applied on the similarity matrix $\hat{M}$. It is clearly shown that the scPEDSSC method demonstrates superior clustering effect to other methods.

**Fig 5:**
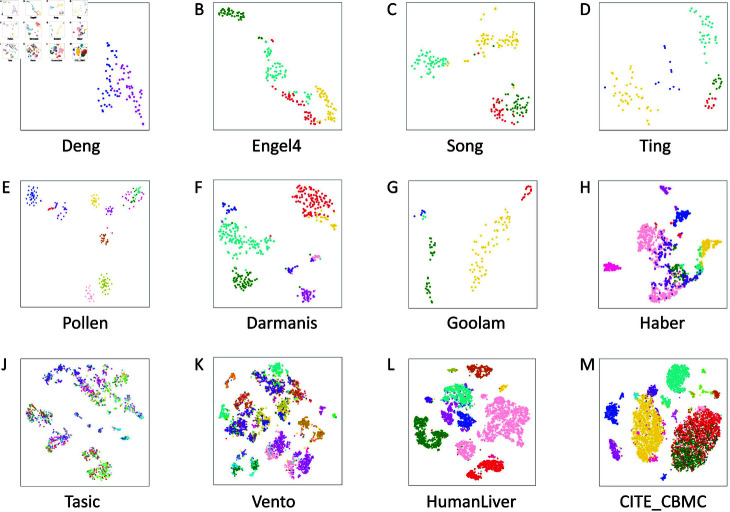
Visualization of clustering results of method scPEDSSC.

Further, the clustering results of method scPEDSSC on the twelve datasets are depicted in Fig 5. It is noticed that Figs 5A–5G display satisfying clustering visualization results, i.e., the clustering number is exactly the same as the actual number of cell-types, and there is less overlap between different clusters. For the rest five datasets with much more cell-types, poor clustering visualization results are presented, as in Figs 5H–5M. The reason may be that with the increase of cell-types, the learned hidden feature information contained in the similarity matrix is insufficient for distinguishing different cell types.

**Fig 6:**
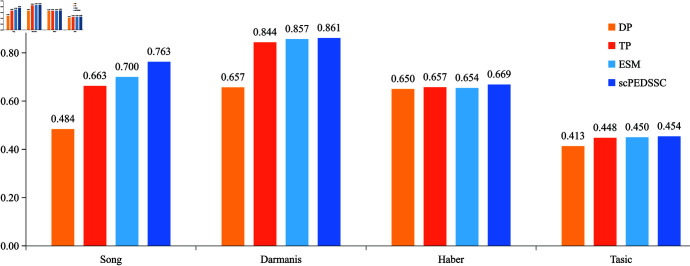
The NMI scores of ablation experiments on four datasets.

### Ablation experiments

**Fig 7:**
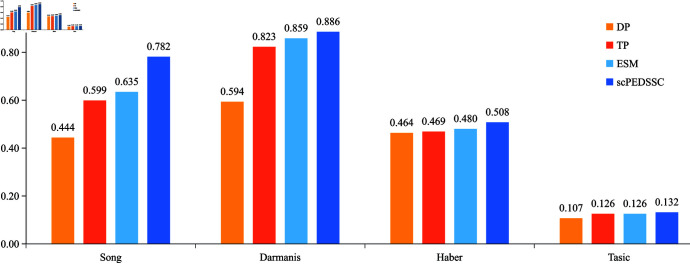
The ARI scores of ablation experiments on four datasets.

In this section, we validate the effectiveness of introducing the Laplace score based data preprocessing, the TPGG distribution, and the enhanced self-expression matrix. Let DP denote the method of replacing “Laplace score based Data preprocessing” with “a conventional preprocessing implemented using the Scanpy Python package,” TP denote the method of replacing the TPGG distribution with the ZINB one, and ESM denote the method of removing the enhanced self-expression matrix. In Fig 6, the NMI scores are compared for the four methods on datasets Song, Darmanis, Haber, and Tasic. From this figure it can be seen that, the scPEDSSC method can acquire the highest NMI score among the comparative ones on each dataset. Taking dataset Darmanis as an example, the NMI scores of methods DP, TP, ESM, and scPEDSSC are 0.6569, 0.8436, 0.8572, and 0.8614, respectively. Fig 7 demonstrates the ARI values of the four methods on the four datasets. The ARI obtained by the scPEDSSC method is still higher than those of the other three ones on the four datasets.

## Conclusion and discussion

The distinguishment of various cells from scRNA-seq data has been regarded as one of the crucial upstream tasks for conducting cell-related studies. In this paper, a deep sparse subspace clustering method scPEDSSC is proposed based on proximity enhancement. It begins with screening genes in terms of Laplace scores. Then it constructs a self-expression matrix from training a deep auto-encoder with adopting the TPGG distribution. The self-expression matrix is further enhanced to produce a similarity matrix for conducting spectral clustering. Twelve real biological datasets were adopted to perform the comparisons among method scPEDSSC and eight state-of-the-art single-cell clustering ones. The experimental results indicate that the proposed method scPEDSSC has better performance than other comparison methods in general.

However, during the process of experiments, it is noticed that the performance of method scPEDSSC is affected negatively by the number of clusters and cells, i.e., the learned hidden feature information is insufficient for distinguishing different cell types when the cluster number or the cell number is large. It may be due to the fact that the probability distribution function cannot model the distributional properties of scRNA-seq data very well. More appropriate probability distribution function should be further devised, which will be studied in a future work.

## Supporting information

S1 TableThe NMI and ARI under different $n_{1}^{e}$, $n_{2}^{e}$, $n_{3}^{e}$, $n_{1}^{d}$, $n_{2}^{d}$, $n_{3}^{d}$ and $l_{r}$ ($\lambda_{1}$=0.2, $\lambda_{2}$=1.0, $\lambda_{3}$=0.5).(XLSX)

S2 TableThe NMI and ARI scores under different $\lambda_{1}$, $\lambda_{2}$, and $\lambda_{3}$ ($n_{1}^{e}$=$n_{3}^{d}$=256, $n_{2}^{e}$=$n_{2}^{d}$=32, $n_{3}^{e}$=$n_{1}^{d}$=10, $l_{r}$=0.001).(XLSX)
